# Metal Detoxification in Land Plants: From Bryophytes to Vascular Plants. STATE of the Art and Opportunities

**DOI:** 10.3390/plants11030237

**Published:** 2022-01-18

**Authors:** Elisa Fasani, Mingai Li, Claudio Varotto, Antonella Furini, Giovanni DalCorso

**Affiliations:** 1 Department Biotechnology, University of Verona, Str. Le Grazie 15, 37131 Verona, Italy; elisa.fasani@univr.it; 2 Department of Biodiversity and Molecular Ecology, Research and Innovation Centre, Fondazione Edmund Mach, Via E. Mach 1, 38010 San Michele all’ Adige, Italy; mingai.li@fmach.it (M.L.); claudio.varotto@fmach.it (C.V.)

**Keywords:** heavy metals, evolution, hyperaccumulation

## Abstract

Potentially toxic elements are a widespread concern due to their increasing diffusion into the environment. To counteract this problem, the relationship between plants and metal(loid)s has been investigated in the last 30 years. In this field, research has mainly dealt with angiosperms, whereas plant clades that are lower in the evolutive scale have been somewhat overlooked. However, recent studies have revealed the potential of bryophytes, pteridophytes and gymnosperms in environmental sciences, either as suitable indicators of habitat health and elemental pollution or as efficient tools for the reclamation of degraded soils and waters. In this review, we summarize recent research on the interaction between plants and potentially toxic elements, considering all land plant clades. The focus is on plant applicability in the identification and restoration of polluted environments, as well as on the characterization of molecular mechanisms with a potential outlet in the engineering of element tolerance and accumulation.

## 1. Introduction

Elements that have a density of more than 5 g/cm^3^ are defined as heavy metals (HMs) [1]. They are present mainly in rock formations, from where they can be released either by natural erosive processes or anthropical intervention; only a limited number of heavy metals are soluble under physiological conditions and thus, bioavailable to living organisms. A certain number of HM is essential for the normal metabolic functioning of organisms (i.e., iron [Fe], molybdenum [Mo], manganese [Mn], zinc [Zn], nickel [Ni], copper [Cu] and cobalt [Co]); both their deficiency and excess can lead to physiological stress due to nutritional imbalance and toxicity. Others (such as arsenic [As], silver [Ag], mercury [Hg], antimony [Sb], cadmium [Cd] and lead [Pb]) have no known biological function and are toxic even when present at low concentration. More recently, the nomenclature *heavy metals* have encountered opposition between researchers, also because some toxic elements, such as As or selenium (Se), are not actual metals, but metalloids or non-metal elements; to overcome this issue, a great variety of definitions have been proposed (for an overview about this topic, refer to an opinion by Duffus [2])). In 2019, Pourret and Hursthouse [3] proposed the term *potentially toxic elements* (PTE) instead of heavy metals in studies concerning environmental science. We will adopt this term throughout the manuscript, therefore, including heavy metals under the chemical point of view, together with metalloids and other elements important for their toxicity towards living organisms; in cases where just genuine heavy metals are considered, we will refer to these with the abbreviation HMs.

Environmental pollution caused by a variety of anthropic activities has substantially increased the presence of PTEs in the biosphere, posing serious threats to all forms of life [4]. PTE contamination can easily reach aquatic systems and soils and subsequently the food chain [5]. Pollution by metals or metalloids not only impacts crop production and quality but also affects atmosphere and water quality and endangers the health and life of plants, animals and human beings. Indeed, when essential PTEs are present in excess or unessential ones are introduced in the body even in small doses, they can cause adverse health effects in both acute and chronic dosing [6]. Symptoms of PTE toxicity in humans include neurological defects, cardiovascular, liver and kidney failure, and, if not promptly recognized as due to PTE exposure, can lead to severe clinical conditions [7,8]. Similarly, when the elevated concentrations of PTE are present in the soil they interfere with plant nutrient uptake, leading to toxicity symptoms and growth inhibition on most plant species [9].

At the cellular level, toxic or excess PTEs can displace and substitute metallic cofactors from enzymes and other proteins, such as transcription factors, altering enzyme activity and the expression patterns of numerous genes; in addition, the imbalance of metal homeostasis leads to damages to lipids, proteins and nucleic acids. The major toxicity route is the production of free radicals and *reactive oxygen species* (ROS), causing oxidative damage [10], followed by the activation of the activity of enzymes that contribute to ROS detoxification [11]. Thus, to minimize the induced damages, plants, as well as other organisms, have developed tightly regulated mechanisms to selectively take up essential elements and use them in metabolic functions, and to avoid or metabolically inactivate toxic/excessive ions. Enzymatic and non-enzymatic defense mechanisms are in charge of ROS control. Enzymatic scavenging of ROS is operated by antioxidants, such as superoxide dismutases (SOD), catalases (CAT), peroxidases (POD) and glutathione reductases as well as non-enzymatic antioxidant molecules including organic acids, glutathione, ascorbic acids, alpha-tocopherols and others [12]. In addition, intracellular and extracellular chelation mechanisms are crucial for PTE detoxification. The binding of PTEs to amino acids, metallothioneins and phytochelatins guarantees that excess or toxic elements are metabolically unavailable and eventually sequestered outside the cytoplasm (e.g., in vacuoles or cell wall) preventing their entrance into energy organelles [13]. Among land plants, different species have evolved various degrees of tolerance to PTEs, with consequent implications in their ability to survive and grow in contaminated areas. A variety of morphological and physiological properties are responsible for the capacity of different species to bind metals on their surfaces or to take them up intracellularly [14]. In this review we summarize actual knowledge on the plant—PTE relationship, taking into consideration the main land plant taxa, i.e., bryophytes and pteridophytes for spore-bearing plants, and gymnosperms and angiosperms as seed-bearing plants.

## 2. Spore-Bearing Plants

### 2.1. Bryophytes

Bryophyte is a class of non-vascular green land plants constituted by around 15,000 species [15]. As revealed by recent phylogenomic reconstructions [16,17], the three lineages of bryophytes (hornworts, mosses and liverworts) likely form a monophyletic clade which is the sister group to tracheophytes. Bryophytes are small, possess both simple morphology and anatomy with a dominant haploid life cycle, and are able to disperse either by spores or by vegetative propagules, such as fragments of leaf and thallus or specialized structures with a high regeneration capacity [15,18,19]. Bryophytes grow in diverse habitats, such as soil, tree trunks, barks and branches, as well as rock surfaces, and take the mineral nutrients required for their growth directly from the substances dissolved in the moist substrates they live on [20]. Due to the absence of epidermal cuticles and the high surface-to-volume ratio, bryophytes are highly susceptible to environmental impacts. In addition, their high ion-exchange capacity, ion chelation and wide geographical distribution make bryophytes excellent biological organisms for monitoring PTE pollution.

Because of anthropogenic activities associated with urbanization and industrial development, more and more non-degradable hazardous PTEs have been released into the environment and entered natural habitats. Among the three lineages of bryophytes, mosses [21,22,23,24] and liverworts [25,26] have been widely explored as biological tools to assess PTE pollution in both terrestrial and aquatic environments. Bryophytes are, in fact, able to accumulate large quantities of HMs over their entire surface, either from the atmosphere or from the substrate, without visible negative impacts on their growth and development [27]. Positive correlations between PTE cellular accumulation and environmental levels have been identified in a range of bryophytes [28], confirming the relevance of these simple plants as biomonitors. Interestingly, there is a great variation in the accumulation of specific PTEs among different species and habitats [29]. For instance, the mosses *Funaria hygrometrica* and *Warnstorfia fluitans* accumulate large quantities of Pb and As, respectively [30,31]. The liverwort *Marchantia polymorpha* accumulates high levels of Cu, Zn and Cd [32,33]. Bryophytes have also the capacity to concentrate rare earth elements [34]. Thus, the evergreen bryophytes allow following the presence of specific elements and their deposition patterns in the respective environment over defined time spans.

Despite their apparent structural simplicity, bryophytes have developed a set of different strategies to tolerate elemental stress. As a general mechanism, they can produce cell wall barriers to prevent PTEs from entering their protoplasts [35]. For example, the moss *Scorpiurum circinatum* was shown to immobilize toxic metal ions in its cell walls [36]; similarly, excessive Zn intake was blocked by the cell wall and plasma membrane in the moss *Pohilia drummondii* [37]. However, the molecular regulation of cell wall biosynthesis and composition still needs further elucidation to understand their role in PTE tolerance in bryophytes. In addition to cell walls, differences in cation exchange capacities and transporter activity in the plasma membrane could generate different elemental tolerance among different species of this clade [38]. Inside the cell, chelation of ions by cysteine-rich oligopeptides glutathione (GSH) and phytochelatins (PCs) is a fundamental approach employed for PTE detoxification; ligand synthesis, accumulation in the cytoplasm and sequestration into vacuoles by ATP-binding cassette (ABC) transporters have been demonstrated to confer tolerance to different extents [39,40]. Although both GSH and PCs have been identified in all three lineages of bryophytes [41], the main contributor to metal chelation is apparently different among the species. At least with regards to Cd, its detoxification is mainly driven by GSH in the case of the moss *Leptodictyum riparium* [42], whereas PCs play essential roles in the liverwort *M. polymorpha* [43]. In addition to ion chelation, the antioxidant defense system, comprising a diverse array of enzymatic and non-enzymatic components, is also an efficient and sophisticated regulator to cope with oxidative stress induced by PTEs [44]. For instance, modulation of the activity of antioxidant enzymes (CATs and PODs) was observed in the *moss Hypnum plumaeforme* [45], as well as in the aquatic bryophyte *Fontinalis antipyretica* subjected to metal exposure [46]. Thus, despite the application of these mechanisms being species-specific, the measurement of intracellular ROS content and/or of antioxidant activity provides an effective overview for the overall intracellular redox state in response to PTE stress.

### 2.2. Pteridophytes

Pteridophytes are a paraphyletic group of plants encompassing ferns and other related clades (lycophytes and monilophytes; [47]), that constitute the second largest group among land plants [48]. Despite the crown age of pteridophytes dating back to about 400 million years before the present [49], the majority of extant pteridophyte species result from a secondary diversification that took place concomitantly to angiosperm diversification [50]. The patterns of adaptive evolution and diversification in extant pteridophytes, therefore, largely parallel those observed in flowering plants [51]. Ferns represent the large majority among the ca. 13,000 species of pteridophytes currently recognized [47], thus constituting a group of primary interest to study the evolution of metal(loid) tolerance and hyperaccumulation in vascular plants and their potential use in phytoremediation [52,53,54,55]. In particular, some aquatic ferns from genera *Salvinia* and *Azolla* (family Salviniaceae) can accumulate with good efficiency a wide range of PTE like Au, Cd, Cr, Cs, Cu, Mn, Ni, Pb, Sr and Zn [56,57,58]; in view of this, they could be useful for the phytoremediation of contaminated wastewaters like industrial effluents and sewage water [53,58,59,60,61,62,63,64].

In the genus *Salvinia*, several species (*S. auriculata*, *S. biloba*, *S. herzogii*, *S. minima*, *S. molesta*, *S. natans* and *S. rotundifolia*) have the capacity to accumulate PTEs in sufficiently high amounts to be used for efficient wastewater depuration (e.g., [65,66,67,68,69,70,71,72,73,74,75]), reaching accumulation ranges of 6000–18,000 mg Kg^−1^ dry weight for Cd, Cr, Cu, Fe, Ni and Pb [57,64,76,77]. Natural variation in the capacity to accumulate PTEs exists among species, as in the case of *Azolla*, another aquatic fern genus with good potential for wastewater phytoremediation. *Azolla caroliniana*, for instance, can accumulate As, Cd, Cr, Hg and Pb in the range between 284–963 mg Kg^−1^, while *A. filiculoides* has been reported to accumulate about 10 times as much Cd, Cr, Cu and Pb (6500–9300 mg Kg^−1^). By contrast, *A. pinnata* is a Pb accumulator (ca. 2700 mg Kg^−1^) but can accumulate amounts of Cr, Cu, Hg and Cd (ca. 210–740 mg Kg^−1^) comparable to those of *A. caroliniana* [64].

Additionally, several species of land ferns have been reported to grow in soils contaminated by a variety of different PTEs and are thus, considered valuable ecological indicators of metal pollution [78]. Recent surveys of fern species that are abundant in mining areas reveal a good potential for the exploitation of some of them (especially from the *Pityrogramma* and *Pteris* genera) for mine rehabilitation and/or metal recovery [79,80,81]. Among the seven orders currently recognized of leptosporangiate ferns (subclass Polypodiidae, the most represented among ferns), [82], metal tolerance and accumulation has been studied most intensively in the Polypodiales order, where representative species from more than 13 genera have been characterized so far [53,64]. The most promising Polypodiales genera for phytoremediation are *Adiantum*, *Asplenium*, *Athyrium*, *Azolla*, *Blechnum*, *Nephrolepis*, *Pellaea* and *Pteris*, which display a high degree of natural variation in their ability to accumulate or hyperaccumulate different metals and the metalloid As. For instance, accumulation of Cd varies from only 4.1 mg Kg^−1^ in *Nephrolepis cordifolia* to 1095 mg Kg^−1^ in *Athyrium yokoscense*; Pb varies between 62 mg Kg^−1^ in *Pteris falcata* to 2040–3464 in *Athyrium yokoscense*; Zn from 216 mg Kg^−1^ in *Blechnum nudum* to 2422 mg Kg^−1^ in *Athyrium yokoscense*; As from 814 mg Kg^−1^ in *Athyrium yokoscense* to 14,500 mg Kg^−1^ in *Pteris vittata* [64].

Among the species investigated, three gained recent and widespread attention for their outstanding accumulation capacity of PTEs, namely *Athyrium yokoscense*, *Pityrogramma calomelanos* and *Pteris vittata*. Among them, *A. yokoscense* is the one with the broadest specificity and overall highest accumulation levels. Indeed, *A. yokoscense* can not only accumulate Cd and Pb to about 1000 mg Kg^−1^ and 10,000 mg Kg^−1^, respectively [83,84], but is also highly tolerant and accumulates Zn and Cu up to more than 9000 mg Kg^−1^ and 3300 mg Kg^−1^ in the roots, respectively [85]. *A. yokoscense* is also an efficient As accumulator, with concentrations of this metalloid reaching up to 922 mg Kg^−1^ and 2192 mg Kg^−1^ in above-ground and below-ground organs, respectively [86]. By comparison, *P. calomelanos* and *P. vittata* display a lower capacity to accumulate metals, but they accumulate As to higher levels than *A. yokoscense* [79,86]. *P. calomelanos* can accumulate large amounts of As (>8000 mg Kg^−1^ dry mass) mostly in its fronds, while in the rhizoids As concentration does not exceed 310 mg Kg^−1^ dry mass [87,88]. Similarly, *P. vittata* commonly accumulates As mainly in above-ground organs in concentrations up to 7500 mg Kg^−1^ without any detectable toxic effects, but even higher concentrations of As in fronds (>22,000 mg Kg^−1^) have also been reported [89,90]. Both bioconcentration factor (BCF; the ratio between As concentration in plant tissues and As concentration in soil) and translocation factor (TF; the ratio between As concentration in fronds and As concentration in roots) of *P. vittata* can reach impressively high values, respectively up to 63 and 25 [91,92]. For these reasons, in recent years *P. vittata* has become a valuable model to elucidate the molecular mechanisms for As hyperaccumulation [93]. Both the diploid sporophyte and the haploid gametophyte of *Pteris vittata* can withstand the major forms of environmental arsenic, arsenate (As(V)) and arsenite (As(III)) [90]. According to a recent model of As hyperaccumulation in *P. vittata* gametophyte cells [94], arsenate is taken up into the cytoplasm by the phosphate transporter PvPht1;3 [95], while arsenite uptake is mediated by Tonoplast Intrinsic Protein 4 (PvTIP4) [96]. Once inside the cell, arsenite is directly transported across the tonoplast membrane by the PvACR3 transporter [97] for long-term storage in the vacuole as free arsenite [98]. On the other hand, cytoplasmic arsenate can undergo two different fates: (i) it can be reduced to arsenite by the arsenate reductase PvACR2 [99], or (ii) converted to 1-arseno-3-phosphoglyerate (1-As-3-PG) by PvGAPC1, an unusual glyceraldehyde 3-phosphate dehydrogenase with a very high affinity for arsenate [94]. The Organic Cation Transporter protein (PvOCT4) transports 1-As-3-PG into cytoplasmic vesicles, where it is reduced to arsenite by PvGSTF1, a glutathione S-transferase with arsenate reductase activity, and/or by PvACR2. Fusion of the vesicles to the tonoplast releases the arsenite into the vacuole [94].

## 3. Seed-Bearing Plants: Gymnosperms and Angiosperms

Progressing with evolution, Spermatophyta, i.e., seed-bearing vascular plants, comprise two sister groups, gymnosperms and flowering plants, angiosperms. If the first represent roughly 1% of total plant diversity and are confined mostly to boreal environments and high-elevation lands, angiosperms account for almost 90% of all plant species and are widespread in all Earth’s ecosystems (the rest are non-seed bearing plants, [100]). On the other hand, the former group can be roughly approximated with conifers, which are the most diverse group worldwide, clustering more than 600 species [100,101].

### 3.1. Gymnosperms

Published literature regarding PTE accumulation in gymnosperms is scarce if compared to angiosperms. The majority of published papers deal with the exploitation of gymnosperms as biological monitors [102]. In this context, as previously mentioned for bryophytes, plants are helpful (*i*) to estimate the actual environmental contamination in a particular site, due to the HMs or radionuclides deposition on leaves, and (*ii*) to record contamination across time, e.g., the temporal evolution of PTE availability in the environment, analyzing metal content in bark sheets [103]. Interestingly, while for some authors utilization of biological material for environmental quality monitoring is rather accepted as reliable and affordable [101], recent works pointed out that the usefulness of coniferous trees as bioindicators of pollution may be debatable, especially for those elements that can act as micronutrients. More robust data can be obtained on non-essential elements, such as Cd, Pb, or Hg, whose absorption and mobility across the plant tissues are limited [103]. The effect of PTEs on gymnosperms has been studied, revealing that also members of this clade are characterized by great variability in terms of sensitivity to metal(loid) ions, depending on the element utilized, the plant species considered and the cultural conditions. Indeed, most experiments have been carried out on a laboratory scale, such as in vitro or hydroponic cultures, analyzing germination and behavior of seedlings or young trees [102]. A range of publications deals with the accumulation of trace elements in needles of plants growing in contaminated areas, highlighting that plant behavior in metal uptake largely depends on the species, the metal considered and the environmental context. As for the latter, the presence of airborne pollution and soil elemental levels influence direct uptake via needles, root absorption and translocation processes [104]. For instance, four-year-old *Pinus sylvestris* plants resulted to be more sensitive to Ni and less to Cu, even though both metals determine injuries on fine roots and needles [105]. In *Picea abies* treated with Cd, Pb, Cu, and Zn, the harshest effects were produced by Cd on germination and by Cu and Pb on growth [106].

Common mechanisms, shared with angiosperm relatives, have been pointed out, such as the activity of antioxidant enzymes; for example, superoxide dismutase induction by excess Zn in *P. sylvestris* [107], or increase in peroxidase activity in *P. abies* grown in soil contaminated with Cd [108] counteract the negative effects due to metal-induced ROS production. The response to PTEs in the growth substrate is highly dependent on the plant species and the metal concentrations applied. For example, treating a *P. abies* cell culture with Cd and As induced the synthesis of GSH-S transferases, enzymes known to be involved in contrasting oxidative damages and membrane lipid peroxidation. Interestingly, Pb treatment does not induce the same effect [109].

Additionally, PCs already mentioned non-protein thiols, are involved in metal ion chelation and detoxification by transport and compartmentalization to specific cell districts, such as the vacuole [110]. The production of PCs in response to treatment with metal ions, such as Cd has been confirmed for a long time in coniferous plants (i.e., *Pinus*, *Abies* and *Picea*), *Gingko biloba* and *Cycas revoluta* [111]. Additionally, Fe, Mn and Pb enhanced PC accumulation in needles of *P. sylvestris* grown in contaminated sites, consistently with the increased amount of metals accumulated in the same tissues [101]. A similar effect in stimulating PC accumulation was reported in *Picea rubens* cell suspensions treated with excess Zn and Cd [112].

Considering degraded soils and the possibility of reclamation by means of reforestation, the understanding of plant tolerance to PTE abundance helps in choosing the right species for reforestation of a particular site. According to this, recent literature has emerged dealing with pollution tolerance and maintenance of fitness upon growth on polluted sites. For instance, Curguz et al., [106] found that seedlings of *P. abies* are particularly tolerant to high soil concentrations of Zn, Cd, Cu and Pb, and the spruce could be selected for reforestation in Serbia, whose subsoil was reported to be contaminated with these metals. Another example considered reclamation of polluted soils due to mining activities in Northern Africa by phytostabilization of metals with ecto-mycorrhized *Pinus halepensis*. Indeed, the presence of ectomycorrhizae significantly reduced root-to-shoot translocation of Zn and Cd, enhancing *P. halpensis* tolerance toward these metals [113]. Coniferous plants are particularly adapted to cold-climate regions. *P. sylvestris*, thanks to its high adaptability, is frequently proposed for remediation of soil polluted by industrial activities, and can, therefore, be suitable for reforestation of degraded boreal lands, even though its ability to tolerate and accumulate PTEs is much lower compared to herbaceous species (see later in the text) [114,115]. Indeed, recent experiments showed that *P. sylvestris* behaves as more sensitive in comparison with angiosperm: root and shoot growth of *P. sylvestris* is inhibited by Pb, Zn, and Cd treatment, which also perturbed plant mineral nutrition [113]. Moreover, germination and seedling growth were also inhibited in *P. sylvestris* challenged with moderate (50 µM) Zn excess, and metal accumulation increased in seedling roots rather than in stems, pointing to a retention of the contaminant in the root [107]. Similarly, in four-week-old hybrid *Larix* (*L. x eurolepis*), double Cd accumulation has been detected in roots compared to shoots [116]. Such accumulation in roots rather than in shoots of gymnosperms has been described frequently [102], pointing to a retention of the excess PTE in the roots to preserve photosynthetic tissues from potentially toxic ion concentrations. The accumulation and compartmentation of toxic ions have evolved as a tolerance strategy and allowed plant growth and reproduction in metalliferous soils (either naturally contaminated or degraded by anthropogenic activities).

### 3.2. Angiosperms and the Evolution of the Hyperaccumulation and Hypertolerance Traits

**Figure 1:**
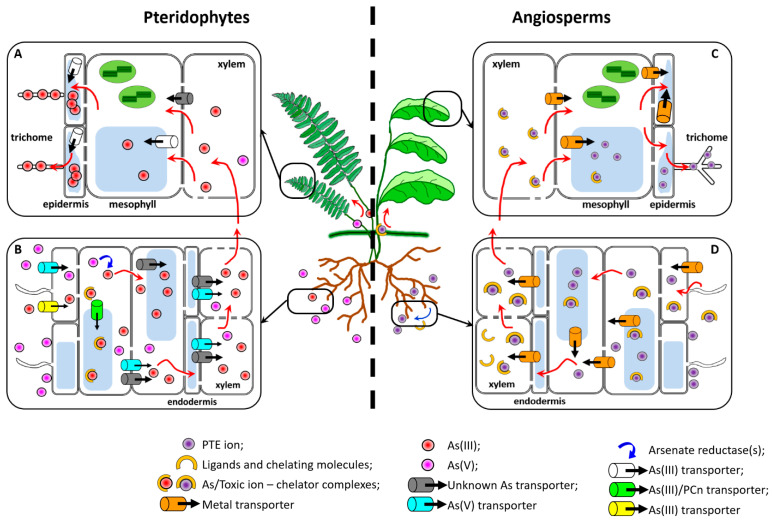
Similarities and differences between hyperaccumulation in ferns and in angiosperm hyperaccumulator plants. In Pteridophytes, As hyperaccumulation is a complex process involving both As(III) and As(V). While As(V) is the major form taken up from the soil, As(III) is the prevalent form transported and stored in the plant. A key process in determining the relative balance of As(III) and As(V) is the reduction of arsenate to arsenite by arsenate reductases in the roots (**B**). As is partly sequestered in the vacuoles both as free As(III) and As(III)/phytochelatin complexes (depicted for simplicity in different cells), but the majority is loaded into the xylem with the contribution of unidentified transporters. (**A**). After unloading from the xylem in the fronds, As(III) is stored in the vacuoles of mesophyll and especially epidermal cells and trichomes, where As concentrations reach the highest values [94,98]. In Angiosperms, the main genetic determinants of heavy metal hyperaccumulation are constitutively overexpressed in hyperaccumulators species. Coded proteins are involved in metal transport and homeostasis. For instance, as highlighted in (**D**), in the root of hyperaccumulator plants a variety of membrane transporters are involved in the transport of metal ions towards the shoot, decreasing root vacular accumulation in favor of an enhanced root-to-shoot transport. Additionally, overproduction of ligands, both intracellularly and secreted into the rhizosphere, (such as Histidine, required for Ni hypertolerance and hyperaccumulation [110]) plays a role as hyperaccumulation determinant, binding to HM ions and adjuvating their transport through the xylem sap. (**C**). Once translocated towards the shoot, by the action of overexpressed vacuolar and plasma membrane transporters, HM ions are sequestered in vacuoles of mesophyll and epidermis cells and in leaf cell walls respectively, and, in some species, such as *Arabidopsis halleri*, in cell trichomes [120]. As mentioned in the text, such sequestration is important to exclude the toxic ions from energy handling organelles, such as chloroplasts and mitochondria.

Adopted by both pteridophytes and angiosperms, worth of note is the evolution of the hypertolerance and hyperaccumulation traits (Figure 1), which are not represented in woody plant species belonging to the gymnosperms [102,117,118]. In angiosperms, due to the relative easiness of intra- and inter-specific crosses, both traits have been extensively studied in experiments with segregating populations derived by crosses between phylogenetically closed species characterized by opposite behavior in terms of metal accumulation and tolerance. Genetic determinants of the two traits, organized in quantitative trait loci, are different; indeed, PTE tolerance can be achieved through two opposite mechanisms. Some plants tend to exclude toxic ions from absorption, thereby limiting the root-to-shoot translocation of the metals to physiological concentrations and are, therefore, tolerant but not accumulators of the PTE. Conversely, other species evolved the ability to accumulate great amounts of PTE in their aerial parts, where the ions are sequestered in apposite cell tissues and/or compartments, removing them from the cytosolic environment [119]. These species are both tolerant and, due to their accumulation ability, defined hyperaccumulators (for a review, see [120]).

Among angiosperms, species able to hyperaccumulate PTE are distributed in many families and genera, showing that the trait has evolved independently many times. Up to now, more than 700 species have been demonstrated to hyperaccumulate one or more PTEs and are listed in the Global Hyperaccumulator Database (http://hyperaccumulators.smi.uq.edu.au/collection/ accessed on 30 December 2021) which is constantly updated. Interestingly, two families are more represented in this database, i.e., Brassicaceae and Phyllanthaceae, and members of these families are currently considered as models for studying metal hyperaccumulation in angiosperms, such as *Arabidopsis halleri*, *Noccaea* spp., *Alyssum* spp.

Interestingly, several genes that take part in metal transport and homeostasis, and those that encode metal chelators and are involved in the stress response, have been correlated with the hyperaccumulation trait; however, these determinants are not specific of hyperaccumulators but are rather constitutively overexpressed in these species, conferring them the ability to tolerate and accumulate in specific plant organs and/or tissues, huge amounts of metal ions [121]. Mechanisms induced in angiosperms to tolerate excess PTE, as well as those enacted by hyperaccumulators, have been extensively studied and reported in the literature [120]. The first examples are the enhanced accumulation of ion chelating compounds, such as histidine or nicotianamine, involved in hyperaccumulation of Ni in *Alyssum* and Zn in *A. halleri* respectively [122,123]. The overexpression of transporter proteins involved in root-to-shoot metal translocation was also correlated to hyperaccumulation. Such overexpression has been reported, in some cases, as the result of genomic DNA expansion and/or promoter modification, driving enhanced mRNA transcription, as in the case of the Zn transporter HMA4 of *A. halleri* [124]. However, plant capacity for PTE tolerance and accumulation cannot be ascribed to a limited number of genetic determinants, but rather to a whole reorganization of developmental, nutritional and metabolic processes [119,125], whose extent is still under study.

## 4. Conclusions and Future Perspectives

Deep knowledge of the interaction between plants and metal ions/metalloids in soil, both toxic or nutrient elements, is an essential base for a successful application of non-vascular and vascular plants for the identification and reclamation of PTE-polluted environments. In addition to the more straightforward approaches, such knowledge allows for manipulation of PTE tolerance and accumulation in target plant species. From a biotechnological point of view, there are two main fields of application of genetic engineering of the plant actors, leading to either improved metal accumulation or exclusion from the cellular environment. Firstly, biotechnology may aim to tailor PTE content in edible plant parts, by enhancing the transport and accumulation of essential metal(oid)s for human nutrition (i.e., biofortification), or reducing the accumulation of toxic elements in crops cultivated on risky lands. Secondly, an increase in the accumulation of toxic elements in above-ground plant tissues is significant for phytoremediation purposes, in view of adopting engineered plants to remove HMs from contaminated soils or waters.

The in-depth insight that is being currently gained regarding the genetics of metal(loid) tolerance and accumulation is a valuable source of genetic information (considering both coding and non-coding sequences) that could be employed to control ion accumulation in particular plant tissues or districts. Recent reviews detail the high number of experiments aimed at modulating metal(oid) accumulation in plants, adopting as a system a variety of model plants, trees and edible crops [126,127]. Moreover, even if most literature concerns flowering plants as “gene donors” (generally overlooking the prokaryotic kingdom as well as spore-bearing plants), attention has recently moved toward pteridophytes. For instance, the *P. vittata* As transporter PvACR3, localized in the tonoplast of the gametophyte, is able to enhance resistance to As-contaminated growth substrates in transgenic *A. thaliana* plants, even though it localizes to the plasma membrane in *A. thaliana* cells [128]. Interestingly, during evolution, the ACR3 gene was lost from the angiosperm genomes [128].

The new breeding techniques, such as CRISPR/Cas-mediated genome editing, enable the targeting of specific sites on the DNA, allowing to precisely modify up to a single nucleotide in the sequence, obtaining highly predictable modifications and transgene-free organisms that could be of particular interest for a concrete application.

## Data Availability

The study did not report any data.
